# Physician Aid in Dying for Dementia: The Problem With the Early vs. Late Disease Stage Distinction

**DOI:** 10.3389/fpsyt.2021.703709

**Published:** 2021-09-27

**Authors:** Marie Elisabeth Nicolini

**Affiliations:** ^1^Katholieke Universiteit Leuven, Center for Biomedical Ethics and Law, Leuven, Belgium; ^2^Department for Bioethics, Clinical Center, National Institutes of Health, Bethesda, MD, United States

**Keywords:** physician aid in dying, assisted suicide, euthanasia, dementia, policy, law, ethics, advance directive

## Abstract

**Background:** Physician aid in dying (PAD) based on dementia is a contentious, highly debated topic. Several countries are considering extending their existing laws to include requests in incompetent patients based on a previously written advance directive. Discussions about this issue often invoke a distinction based on disease stage. The Dutch practice uses this distinction in classifications of dementia PAD cases and in guidance for clinicians. This paper explores the problem with this distinction for assessments of persons at the margins of competence.

**The Problem:** Dutch guidance for clinicians uses an early vs. late-stage disease distinction to refer to requests from competent and incompetent persons. However, the use of disease stages is problematic, both conceptually and empirically. Conceptually, because it goes against very functional model of competence that guidance recognizes. Empirically, because it creates problems for classifying and evaluating patients at the margins of competence.

**Possible Ways Forward:** Classification of cases and guidance should be based on competence, not disease stage. This requires rethinking decision-making for patients with dementia. Several possibilities are described, ranging from redefining the scope and role of advance directives in this context to exploring different types of decision-making frameworks.

## Introduction

Physician aid in dying (PAD) based on dementia is a contentious topic that has gathered significant attention over the past decade. The practice is permitted in some European countries, though with significant differences across jurisdictions. For example, the Netherlands is so far the only country permitting PAD for dementia in persons who are not competent, based on a previously written advance directive. A recent high-profile court case in the Netherlands lead to extensive debates about the use and scope of advance directives in this context (1–3). The recent Spanish law allows for advance requests, Canada is currently considering their inclusion in its law and in Belgium, proposals exist for expanding the law to include such requests (4–6). Often, in debates about whether to expanding access to PAD for persons who are incompetent, a distinction is invoked based on disease stage. A similar distinction is used in the Dutch practice for classifying dementia PAD cases and in guidance for clinicians (7, 8). This paper will argue that this distinction based on disease stage creates conceptual confusion and insufficiently clear guidance for evaluating persons “at the margins of competence,” i.e., cases where it is unclear whether competence is retained or impaired. Given that competence is an essential component of a valid informed PAD request, and given that PAD for dementia is a highly complex, ethically fraught topic involving difficult clinical and ethical judgments, its policy and guidance should provide clear and sound support for clinicians involved in these assessments. After describing the problem, this paper will explore possible ways for rethinking decision-making in this context.

## The Problem With the Distinction Based on Disease Stage

The Dutch law (*Termination of Life on Request and Assisted Suicide Act*) makes a distinction about whether or not to invoke an advance directive based on the person's competence (7). The law contains two sections (Box 1). Section 2.1 states the six legal requirements that apply for a competent patient's request to be considered eligible (so-called concurrent request). Section 2.2 states that a “patient aged 16 or over who is decisionally competent may draw up an advance directive, setting out a request for euthanasia (so-called advance request). If at some point the patient is no longer capable of expressing their will, the physician may accept the advance directive as a request pursuant to section 2 (1)(a) of the Act.” Hence, in a patient who is no longer competent, a physician can use the patient's previously written advance euthanasia directive (commonly abbreviated as AED) to meet the requirement that the request be voluntary and well-considered. In the case of an AED, section 2.2 of the Act states that the due care criteria, as described under section 2.1, apply “mutatis mutandis” (7).

Box 1 **The Dutch law (Termination of Life on Request and Assisted Suicide Act)**

Section 2.1. The physician must:

(a) be satisfied that the patient's request is voluntary and well considered;
(b) be satisfied that the patient's suffering is unbearable, with no prospect of improvement;
(c) have informed the patient about their situation and prognosis;
(d) have come to the conclusion, together with the patient, that there is no reasonable alternative in the patient's situation;
(e) have consulted at least one other, independent physician, who must see the patient and give a written opinion on whether the due care criteria set out in (a–d) have been fulfilled;
(f) have exercised due medical care and attention in terminating the patient's life or assisting in his suicide.

Section 2.2. A patient aged 16 or over who is decisionally competent may draw up an advance directive, setting out a request for euthanasia. If at some point the patient is no longer capable of expressing their will, the physician may accept the advance directive as a request pursuant to section 2 (1)(a) of the Act.

To interpret the voluntary and well-considered request requirement in concurrent requests (i.e., non-AED based), clinicians would turn to the EuthanasiaCode, i.e., the guidance issued by the Dutch Euthanasia Review Committees (RTE). The Code states that the request has to be voluntary, the patient must be decisionally competent and the request must be well-considered (7). The focus here is on competence in concurrent requests. Competence is defined in the Code according to the so-called functional model (7, 9). That is, to be considered competent, a person should show all four of the following functional abilities: the ability (a) to understand, (b) to reason, (c) to appreciate how the decision applies to them, and (d) to communicate their choice (9). The assessment of competence is in practice, an all-or-nothing decision: one either has competence or not (10). The problem is that the EuthanasiaCode, in the sections about the evaluations of PAD requests based on dementia, provides guidance by making a distinction based on specific diagnostic disease stages, i.e., early vs. late stage dementia (7). That is, those who are “decisionally competent” in relation to their request fall under the category early-stage dementia. Those who are “no longer decisionally competent” fall under the category late-stage dementia.

The RTE's pairing of categories of competence with diagnostic disease categories is problematic from a conceptual and from an empirical point of view. From a conceptual perspective, this is not how the functional model of competence is supposed to work. The very purpose of this model is to provide a *functional* framework for assessing competence based on a person's functional cognitive abilities, regardless of diagnostic classifications. This is important because it is a contradiction to endorse the functional model of competence, if de facto some type of status-based approach is used, where competence is associated with the presence of a disorder, or a particular stage of the disorder.

From an empirical perspective, there are indications that this confusion has downstream implications in practice, for example, for the way dementia PAD cases are reviewed and classified by the RTE. A vast majority of cases reported to the RTE are classified as early stage: for example, in 2019, out of the 162 reported Dutch dementia PAD cases, only two were classified as late stage and 160 were instead classified as early stage (8). Similar discrepancy exists for other years. But in a chronic and progressive condition like dementia, a significant portion of cases fit neither label of very early or very late disease stage. Rather, they fall somewhere in between these two extremes. In these cases, the disease stage does not map neatly onto the competence categories: that is, patients might be categorized as early and competent in relation to their request, but de facto be incompetent.

Empirical research on the Dutch practice has shown that this indeed occurs and has given an indication of how large this group may be. A content analysis of 59 cases classified as early stage showed that in about a *third* of cases there was a strong indication of incompetence, either because one of the physicians involved indeed deemed a patient incompetent, or because they needed to resort to non-verbal clues, existing AEDs, or previous statements to determine the patient's competence (11). In these cases, physicians deviated from the standard guidance of using the functional model of competence, either by lowering the threshold or by according more weight to the patient's prior values rather than to their functional abilities (12). More empirical research is needed to gain further insight into these decision-making processes. But available emerging evidence suggests that the problem cannot be dismissed.

The stage-based distinction risks disregarding the complex reality of the substantial portion of patients who are at the so-called margins of competence, that is, patients whose level of competence is unclear. These patients may, for example, have a retained ability to communicate, but without meeting the bar for competence. In other words, “the various significant capacities can come apart” in some cases, complicating competence assessments (10). The difficulty is that these persons, while lacking competence according to the standard definition, “are not yet at the point where their advance directive have the greatest authority” (10). This raises important questions about how they should be assessed in the context of PAD. The current Dutch practice considers these cases under the umbrella of early stage and competent, potentially overlooking important policy discussions about how these persons should be evaluated. The empirical evidence that a substantial portion of patients is misclassified by the RTE, is a first warning sign that current decision-making frameworks might be inadequate or insufficiently tailored to the needs of these persons.

This is important because competence is a key requirement of the informed consent doctrine, together with information and voluntariness (13), and is, as such, crucial for a valid informed PAD request. While competence is not sufficient for a valid and informed request, its importance as a key component warrants attention in and of itself. To ensure that a concurrent request is valid, there needs to be a clear and sound framework to assess, review and categorize persons of all competence levels who request and receive PAD, including those at the margins of competence. This is especially relevant because, given the progressive nature of the disease, these complex cases are not rare. The problems with pairing levels of competence with disease stage warrant further discussion and point to the need to clarify what decision-making model should be used.

## Possible Ways Forward

The disease stage-based distinction in itself is intuitive and could be meant to provide conceptual clarity for policy and practical purposes. However, the conceptual and empirical issues with using dementia disease stages *as a proxy* for competence suggests that the use of the disease stage distinction to guide, review, and categorize cases should be rejected, and replaced by guidance based on competence. This would allow for drawing adequate attention to the question of how to address requests from patients who are at the margins of competence, without the unhelpful reference to their disease stage. This will require reconceptualizing decision-making in persons with dementia, allowing for a more solid and consistent framework for clinicians that clarifies expectations and roles of all parties involved. Drawing on decision-making frameworks that are used in other end-of-life contexts, possible ways for carrying out this solution are described below. The options described are not exhaustive, but rather, are meant as a starting point for further discussion.

### Expand the Scope of AEDs

A first option would be to invoke section 2.2 for all cases in which a person does no longer meet the bar for competence, provided the person has a previously written AED. This would avoid categorizing persons as competent when they are not. But such a reading could be at odds with the purpose of this clause of the law, presumably intended for persons who are clearly *incompetent* at the time of their request.

The main task here would be to define how we interpret the clause that the person is “no longer capable of expressing their will.” This can be interpreted as incompetent or as unable to communicate. The difficulty is that the RTE guidance on PAD for dementia recommends invoking an AED for patients who are “no longer decisionally competent” *and* “no longer able to communicate.” But these are two very different concepts: the ability to communicate is only one of the four necessary abilities required for competence according to the functional model. Certainly then, the two cannot be considered interchangeably. Therefore, for a broader interpretation of this legal clause to work, and for an AED to be invoked in all cases of incompetence, including those at the margins of competence, guidance would need to first clarify what exactly “expressing one's will” means.

### Allow for the Use of AED as a Decision-Making Aid

A second option would be to allow for a person who does not meet the bar for competence to use the previously written AED as additional tool for decision-making (14). The AED would then function as a decision aid or support mechanism, compensating for the person's impaired cognitive abilities and assisting the person in their request. Similarly, some have suggested that an AED should be used flexibly (15). However, it is important to clarify what such a flexible use would look like. If it means altering the content of the AED, or interpreting the content in light of the context, this would seem to defeat its very purpose. This is perhaps what some mean when they state that using AEDs as a tool seems “counterintuitive” (1). If instead the proposed flexibility refers to the time point for when the AED should be invoked, the question is whether an AED is the right instrument for this type of decision-making in patients who are at the margins of competence.

### Adopt Another Decision-Making Type Model

The option of substituted decision-making by a surrogate is rarely discussed within the context of PAD, but it is, in theory, a possibility to consider. Moreover, current practice already involves more physician input than a strict functional model of competence would allow for. For example, physicians' reliance on previous conversations with the patient or their interpretation of non-verbal and bodily cues (11) can be seen as a form of substituted judgment. Allowing for substituted judgment by a surrogate decision-maker designated by the person, would have the advantage of being clearly defined and regulated as such. Substituted decision-making is typically not considered an option in the context of PAD. But to the extent that some form of substituted judgment is already present in complex cases, the different stakeholders' roles need to be made explicit.

A final option would to resort to a supported decision-making model, a model which is gaining traction around the world (16). Different interpretations exist: for example, the UN Convention on the Rights of Persons with Disabilities endorses a more radical interpretation, granting all persons legal capacity regardless of disability or decision-making skills, and calling for abolishing all forms of substitute decision-making (17). A different model, endorsed in several US states and elsewhere, instead views it as complementary to the existing framework, defining supported decision-making as an agreement between a person and a supporter who assists the person in making decisions (18). The type of support needed, and the areas of cognitive function for which support would be invoked, can be determined and consolidated through an agreement. This approach aims at maximizing the person's self-determination, while clarifying roles and expectations for all involved parties. Although these emerging frameworks have their own unresolved challenges and limitations, it is worth exploring what their role could be in the context of PAD for dementia.

## Conclusion

PAD for dementia a global, controversial topic raising significant ethical and clinical questions. Some countries, who already allow PAD for non-terminal disorders like Belgium and Canada, are actively discussing allowing the use of advance directives for PAD in incompetent patients, as is permitted in the Netherlands. This paper argued that the early vs. late disease distinction often invoked in debates as well as in the Dutch practice, provides insufficient clarity as to how competence should be assessed, particularly for those at the margins of competence. This is something that countries debating expansion of their PAD laws should take into consideration. Rather than basing classification and guidance on disease stage, it should be based on the level of competence. This requires rethinking decision-making for incompetent patients with dementia in the Netherlands, regardless of their disease stage. In particular, it requires that attention be paid to how this problem should be addressed in practice. Hopefully, the taskforce on dementia PAD of the Royal Dutch Medical Organization (KNMG), whose report is due to come out later this year, will shed light on this important question.

## Data Availability Statement

The original contributions presented in the study are included in the article/supplementary material, further inquiries can be directed to the corresponding author/s.

## Author Contributions

The author confirms being the sole contributor of this work and has approved it for publication.

## Conflict of Interest

The author declares that the research was conducted in the absence of any commercial or financial relationships that could be construed as a potential conflict of interest.

## Publisher's Note

All claims expressed in this article are solely those of the authors and do not necessarily represent those of their affiliated organizations, or those of the publisher, the editors and the reviewers. Any product that may be evaluated in this article, or claim that may be made by its manufacturer, is not guaranteed or endorsed by the publisher.
